# Regulatory Consideration for the Nonclinical Safety Assessment of Gene Therapies

**DOI:** 10.1089/hum.2022.090

**Published:** 2022-11-14

**Authors:** Jeffrey S. Moffit, Diann L. Blanset, Jessica L. Lynch, Timothy K. MacLachlan, Kathleen E. Meyer, Rafael Ponce, Laurence O. Whiteley

**Affiliations:** ^1^ Carbon Biosciences, Lexington, Massachusetts, USA.; ^2^ Boehringer Ingelheim, Ridgefield, Connecticut, USA.; ^3^ Janssen Research and Development, Spring House, Pennsylvania, USA.; ^4^ Novartis Institutes for Biomedical Research, Cambridge, Massachusetts, USA.; ^5^ Sangamo Therapeutics, Inc., Richmond, California, USA.; ^6^ Shape Therapeutics, Seattle, Washington, USA.; ^7^ Pfizer, Cambridge, Massachusetts, USA.

**Keywords:** gene therapy, nonclinical, safety, regulatory, ICH, toxicity

## Abstract

The nonclinical safety assessments for gene therapies are evolving, leveraging over 20 years of experimental and clinical experience. Despite the growing experience with these therapeutics, there are no approved harmonized global regulatory documents for developing gene therapies with only the ICH (International Council for Harmonization of Technical Requirements for Pharmaceuticals for Human Use) S12 guidance on nonclinical biodistribution currently under discussion. Several health authorities have issued guidance over the last 15 years on the nonclinical safety aspects for gene therapy products, but many of the recommendations are limited to high-level concepts on nonclinical safety aspects or altogether silent on key topics. Historically, this approach was appropriately vague given our relatively small dataset of nonclinical experience, where a comprehensive and detailed regulatory guidance approach was unlikely to be appropriate to address all scenarios. However, harmonization of key considerations and assumptions can provide a consistent basis for developing the appropriate nonclinical safety development plans for individual programs, reducing uncertainty across regulatory regions and unnecessary animal use. Several key areas of nonclinical safety testing are nearing maturation for a harmonized approach, including species selection, certain aspects of study design, study duration, and unintended genomic integration risks. Furthermore, several emerging topics are unaddressed in current regulatory guidance for gene therapy products, which will become key areas of differentiation for the next generation of therapeutics. These topics include redosing, juvenile/pediatric safety, and reproductive/developmental safety testing, where relevant experience from other modalities can be applied. The rationale and potential study design considerations for these topics will be proposed, acknowledging that certain aspects of gene therapy development are not considered appropriate for harmonization. This article provides an overview of the current nonclinical safety regulatory landscape, summarizes typical nonclinical safety study designs, highlights areas of uncertainty, and discusses emerging topics that warrant consideration. Specific recommendations and perspectives are provided to inform future regulatory discussions and harmonization efforts.

## INTRODUCTION

Gene therapies represent a broad family of modalities that modify or correct abnormalities in genes through the expression (transcription or translation) of transferred genetic materials, and/or through selective alteration of the target genome, or through selective modification of gene expression. This class of genetic medicines has grown to include broad categories of gene delivery or modification of cells *ex vivo* and *in vivo*. The *ex vivo* approaches comprise delivery to specific cell populations, including hematopoietic stem and progenitor cells, T cells, regulatory T cells, and will likely expand. The *in vivo* approaches range from targeted delivery to specific compartments (*i.e.*, subretinal, intrathecal) to systemic administration. These diverse targets are supported by an equally diverse set of delivery modalities, including direct administration, electroporation, viral vectors, and nonviral particles. With a growing number of approved *in vivo* and *ex vivo* gene therapy products globally and more than 1,300 in development, innovators and regulatory authorities are gaining valuable experience informing future gene therapy product development.^1^

Despite the interest in developing gene therapies, there are no approved harmonized global regulatory documents for developing gene therapies. Currently, the International Council for Harmonization of Technical Requirements for Pharmaceuticals for Human Use (ICH) has issued three consideration documents related to virus/vector shedding, oncolytic viruses and germline integration, and a draft harmonized guidance document on biodistribution (ICH S12). Additional ICH guidances include concepts and approaches that could be leveraged for gene therapy development, such as ICH S6 and M3(R2), but are not considered in scope.

Regional regulatory guidances specific to gene therapies have emerged, encompassing general considerations, and modality- and disease-specific recommendations. Because regional guidances lack global harmonization or are case by case in nature, a regulatory environment exists where there is a lack of consistency or predictability for innovators, as evidenced by a recent industry survey on developing gene therapies.^2^ This creates uncertainty in the regulatory acceptability of specific strategies, leading to additional regulatory interactions, conflicting regulatory requirements, unnecessary increased number or length of studies, greater animal use, and longer development times with a questionable benefit to patients. However, given the broad range of gene therapies and the relatively small experience with these products, a comprehensive and detailed regulatory guidance approach is unlikely to be appropriate to address all scenarios, particularly as the underlying molecular and analytical technologies rapidly evolve.

Thus, there is an opportunity to meaningfully harmonize the regulatory expectations for the manufacture, nonclinical, and clinical development of gene therapies under ICH, provided there is flexibility in areas that lack clear consensus. A summary of all major gene therapy guidelines and topics covered are listed in Table 1.

**Table 1.** Overview of global gene therapy guidance and key nonclinical safety topics

Ref, Region, Date	Guideline Title	Modalities				Key Nonclinical Safety Topics Covered								
		Cell Edit	Gene Edit	Nonviral	Viral	Animal Model	DART	Germline Transfer	ADA	DMPK BioD	Genomic Integration	Genomic Editing	CMC Safety	Env Risk/Shedding
A, EMA, 2012, 2021	Guideline on quality, nonclinical, and clinical aspects of medicinal products containing genetically modified cells	Yes	Yes	Yes	Yes	Yes	N/A	Yes	N/A	Yes	Yes	Yes	Yes	Yes
B, EMA, 2019	Guideline on quality, nonclinical, and clinical requirements for investigational advanced therapy medicinal products in clinical trials	N/A	Yes	Yes	Yes	Yes	Yes	Yes	N/A	Yes	Yes	Yes	Yes	Yes
C, EMA, 2018	Guideline on the quality, nonclinical, and clinical aspects of gene therapy medicinal products	Yes	Yes	Yes	Yes	Yes	Yes	Yes	Yes	Yes	Yes	Yes	Yes	Yes
D, EMA, 2008	Guideline on scientific requirements for the environmental risk assessment of gene therapy medicinal products	N/A	N/A	N/A	Yes	Yes	N/A	N/A	N/A	Yes	N/A	N/A	N/A	Yes
E, EMA, 2008	Guideline on the nonclinical studies required before first clinical use of gene therapy medicinal products	Yes	N/A	Yes	Yes	Yes	Yes	Yes	N/A	Yes	Yes	N/A	N/A	Yes
F, EMA, 2007	Guideline on nonclinical testing for inadvertent germline transmission of gene transfer vectors	N/A	Yes	Yes	Yes	Yes	Yes	Yes	N/A	Yes	Yes	N/A	N/A	N/A
G, EMA, 2008	Guideline on safety and efficacy follow-up risk management of advanced therapy medicinal products	Yes	Yes	N/A	N/A	N/A	N/A	Yes	Yes	N/A	Yes	Yes	Yes	Yes
H, EMA, 2009	Q&A on gene therapy	Yes	Yes	N/A	Yes	N/A	N/A	N/A	N/A	N/A	N/A	N/A	Yes	Yes
I, EMA, 2010	Reflection article on quality, nonclinical, and clinical issues related to the development of recombinant adeno-associated viral vectors	N/A	Yes	N/A	Yes	Yes	N/A	Yes	Yes	Yes	Yes	N/A	Yes	Yes
J, EMA, 2019	Questions and answers Comparability considerations for Advanced Therapy Medicinal Products (ATMP)	Yes	N/A	N/A	N/A	N/A	N/A	N/A	N/A	N/A	N/A	N/A	Yes	N/A
K, EMA/ICH, 2009	ICH considerations—General principles to address virus and vector shedding	N/A	N/A	N/A	Yes	Yes	N/A	N/A	N/A	Yes	N/A	N/A	Yes	Yes
L, EMA/ICH, 2006	ICH considerations—General principles to address the risk of inadvertent germline integration of gene therapy vectors	N/A	Yes	N/A	Yes	N/A	N/A	Yes	N/A	Yes	Yes	Yes	N/A	N/A
M, EMA/ICH, 2009	ICH considerations for oncolytic viruses	N/A	N/A	N/A	Yes	Yes	N/A	Yes	Yes	Yes	N/A	N/A	N/A	Yes
N, FDA, 2019	Immunogenicity testing of therapeutic protein products—Developing and validating assays for antidrug antibody detection	N/A	N/A	N/A	N/A	N/A	N/A	N/A	Yes	Yes	N/A	N/A	N/A	N/A
O, FDA, 2006	Gene therapy clinical trials—Observing subjects for delayed adverse events	N/A	Yes	N/A	Yes	Yes	N/A	Yes	N/A	Yes	Yes	N/A	N/A	N/A
P, FDA, 2018	Long-term follow-up after administration of human gene therapy products	N/A	Yes	Yes	Yes	Yes	N/A	N/A	N/A	Yes	Yes	Yes	N/A	N/A
Q, FDA, 2018	Human gene therapy for hemophilia	N/A	Yes	N/A	Yes	Yes	Yes	N/A	Yes	Yes	Yes	Yes	Yes	Yes
R, FDA, 2018	Human gene therapy for retinal disorders	N/A	Yes	N/A	Yes	Yes	N/A	N/A	N/A	Yes	Yes	Yes	Yes	N/A
S, FDA, 2020	Chemistry, manufacturing, and control (CMC) information for human gene therapy investigational new drug applications (INDs)	Yes	Yes	Yes	Yes	N/A	N/A	N/A	N/A	N/A	Yes	Yes	Yes	N/A
T, FDA, 2020	Human gene therapy for rare diseases	N/A	N/A	N/A	Yes	Yes	Yes	N/A	Yes	Yes	N/A	N/A	Yes	Yes
U, FDA, 2018	Testing of retroviral vector-based human gene therapy products for replication competent retrovirus during product manufacture and patient follow-up	N/A	N/A	N/A	Yes	N/A	N/A	N/A	N/A	N/A	N/A	N/A	Yes	N/A
V, FDA, 2021	Interpreting sameness of gene therapy products under the orphan drug regulations	Yes	N/A	N/A	Yes	N/A	N/A	N/A	N/A	N/A	N/A	N/A	Yes	N/A
W, FDA, 2019	Expedited programs for regenerative medicine therapies for serious conditions	Yes	N/A	N/A	N/A	Yes	N/A	N/A	N/A	N/A	N/A	N/A	Yes	N/A
X, FDA, 2013	Preclinical assessment of investigational cellular and gene therapy products	Yes	Yes	Yes	Yes	Yes	Yes	Yes	N/A	Yes	Yes	Yes	Yes	Yes
Y, FDA, 2015	Design and analysis of shedding studies for virus or bacteria-based gene therapy and oncolytic products	N/A	N/A	N/A	Yes	N/A	N/A	N/A	N/A	Yes	N/A	N/A	N/A	Yes
Z, FDA, 2015	Considerations for the design of early-phase clinical trials of cellular and gene therapy products	N/A	N/A	N/A	Yes	Yes	Yes	N/A	Yes	Yes	Yes	N/A	Yes	Yes
AA, FDA, 2015	Determining the need for and content of environmental assessments for gene therapies, vectored vaccines, and related recombinant viral or microbial products	N/A	N/A	N/A	Yes	N/A	N/A	N/A	N/A	N/A	N/A	N/A	Yes	Yes
AB, FDA, Draft2021	Human gene therapy for neurodegenerative diseases	N/A	N/A	N/A	Yes	Yes	Yes	N/A	Yes	Yes	N/A	N/A	Yes	Yes
AC, FDA, Draft2022	Human gene therapy products incorporating human genome editing	Yes	Yes	Yes	Yes	Yes	N/A	Yes	N/A	Yes	Yes	Yes	Yes	N/A
AD, PMDA, 2019	Guideline on quality and safety assurance of gene therapy products	Yes	Yes	Yes	Yes	Yes	Yes	Yes	Yes	Yes	Yes	N/A	Yes	Yes
AE, ANVISA (Brazil), 2020	Interfarma, Resolution of the Board of Directors, RDC No. 338, Provides the registration of advanced therapy product and makes other arrangements.	Yes	Yes	N/A	Yes	N/A	Yes	Yes	N/A	Yes	Yes	N/A	Yes	Yes
AF, Minzdrav (Russia), 2018	Order of the Ministry of Health of the Russian Federation of 08.08.2018 No. 512н “On approval of the Rules of Good Practice for working with biomedical cellular products”	Yes	Yes	N/A	N/A	Yes	Yes	N/A	Yes	Yes	Yes	N/A	Yes	N/A
AG, S. Korea, 2017	Guide-0819-01: Guideline on nonclinical study assessment of gene therapeutics	Yes	Yes	Yes	Yes	Yes	Yes	Yes	Yes	Yes	Yes	Yes	N/A	Yes
AH, S. Korea, 2018	Guideline on quality assessment for gene editing-based advanced therapy medicinal products	Yes	Yes	Yes	Yes	N/A	N/A	N/A	N/A	N/A	Yes	Yes	Yes	N/A
AI, China, 2021	Technical guidelines for nonclinical study and evaluation of genetically modified cell therapy products	Yes	Yes	N/A	N/A	Yes	N/A	N/A	Yes	Yes	Yes	Yes	N/A	N/A
AJ, China, 2017	CFDA Notification 2017/216: Technical guidelines for research and evaluations of cell therapy products (Interim)	Yes	Yes	N/A	Yes	Yes	Yes	N/A	Yes	Yes	Yes	N/A	Yes	N/A
AK, ICH, Draft2022	ICH S12: Biodistribution considerations for gene therapy products	Yes	Yes	Yes	Yes	Yes	Yes	N/A	Yes	Yes	N/A	N/A	Yes	N/A

N/A, not addressed; Yes, addressed or mentioned in the respective guidelines.

DART, developmental and reproductive toxicology; ICH, International Council for Harmonization of Technical Requirements for Pharmaceuticals for Human Use.

This article provides an overview of the current nonclinical safety considerations for gene therapies, summarizes typical nonclinical safety study designs, highlights areas of uncertainty, and discusses emerging topics that warrant consideration. Given the breadth of this topic, the concepts are focused primarily on gene therapies and gene editing products administered *in vivo*. Some concepts and transferable experience from *ex vivo* gene therapies are noted throughout, but in general, *ex vivo* gene therapies are beyond the scope of this article. Specific recommendations and perspectives are provided to inform future regulatory discussions and harmonization efforts.

## CURRENT NONCLINICAL SAFETY CONSIDERATIONS

### General study design requirements

The design and conduct of nonclinical studies are critical to identify, characterize, and ultimately mitigate potential local and systemic toxicities; select a safe initial starting dose, dose-escalation scheme, and dosing regimen; and inform on subject eligibility and clinical monitoring strategies. Study designs will vary based on the product attributes, including, but not limited to local versus systemic delivery, activity, and function of the transgene product, integrating versus nonintegrating vectors, and disease-specific considerations.

Study duration is an important and vaguely described aspect for gene therapies. The nonclinical program needs to capture potential acute, chronic, and/or delayed onset toxicities, as well as potential for resolution of toxicity (Table 1, Guidance C). Due to the differences in therapeutic approaches, modalities, and delivery methods (*e.g.,* local vs. systemic), study duration goals are currently considered on a case-by-case basis. Although the justification for study duration should be based on gene therapy characteristics such as persistence of the vector, immunogenicity, and expression of the transgene, there is an opportunity to harmonize guidance in this area.

The use of biodistribution and pharmacokinetic/pharmacodynamic profiles is the first step in determining study durations. Different modalities, routes of administration, and viral vectors have different distribution and transduction profiles in both the target and off-target tissues, including vector and transgene persistence as well as differences between species. Using adeno-associated virus (AAV) as an example, different serotypes have different biodistribution and transduction rates in different organs and species. Regardless of vector serotype, route of administration, or type of transgene product, steady state is typically achieved within ∼4 weeks of AAV administration.^3^ Steady state will likely be vector specific but understanding the steady state and vector biodistribution should drive the study duration. Based on this, toxicities of a standard viral vector class that reaches steady state of transgene expression within ∼4 weeks should be adequately characterized by a 13-week study. This study duration is supported by ICH S6(R1), where the duration of animal dosing has generally been 1–3 months for most biotechnology-derived pharmaceuticals.

In some cases where novel vectors are being used, there is unknown long-term transgene pharmacology, a longer ramp-up to maximum/steady-state levels of expression, or existing knowledge of the potential liabilities related to transgene pharmacology or the delivery system, IND-enabling studies may need to be longer. However, in alignment with ICH S6(R1), studies should not need to exceed 6 months in length. Unless new data suggest a need to investigate toxicities beyond 3 or 6 months, these studies could also suffice for registration.

### Species selection consideration

Emerging experience indicates that animal model dose–response data may not directly translate to humans and can serve only as a guide.^4^ Regulatory guidance documents from both the EMA and FDA have consistently indicated the need to justify species selection based upon preliminary studies and relevance to predicting the human dose-related pharmacology and toxicology profiles (Table 1, Guidance C, X). The model system(s) needs to consider the delivery vehicle (*e.g.,* viral capsid, liposome, polymer nanoparticle, targeting ligand) and biologic activity of the expressed payload (*i.e.,* transgene or therapeutic gene sequence). Consequently, the species selection needs to consider the ability to predict the human dose-related response of both tissue uptake of the delivery vehicle and the payload biologic activity.

Preexisting and adaptive immunity to the vector capsid and/or the transgene product needs to be considered. Preexisting neutralizing antibodies to a vector's capsid can prevent transduction of tissues and animals should be screened and selected based on their preexisting antibody status. This is particularly a concern for nonrodent studies where preexisting antibodies are more prevalent, particularly for AAV vectors. It has been well established that systemic administration of AAVs to organs such as the liver are substantially affected by the presence of preexisting titers, and some have suggested the same is true for other organ systems, which may also predict safety issues.^5–7^ However, it is also well established that different assays for preexisting titers will yield different results,^8^ as such it is not possible to suggest a specific “cutoff” for titers to be deemed “acceptable”—nor is it possible to in many cases to identify enough animals for *in vivo* studies that are below the sponsor's ideal “cutoff.” Therefore, for some gene therapies, it may be sufficient to prescreen animals and randomize positive animals across dose groups.

Adaptive humoral immune responses to the transgene product may limit the ability to assess the intended pharmacology or adverse pharmacology and may require the use of immune suppression or a species-specific transgene that does not induce an immune response. Adaptive humoral immune response to a secreted transgene could result in antigen–antibody complex-related pathology. Cell-mediated immune response to both the vector capsid as well as transgene product may also lead to the loss of transduced cells that abrogates the desired pharmacology or adverse tissue injury. In these instances, immune suppression may be warranted.

The impact of disease pathophysiology on biodistribution and pharmacologic or toxicologic response should also be considered in designing the nonclinical program to support clinical trials. Additionally, if a delivery device is used, the species selected needs to accommodate this device and the anatomical structures that the device encounters should be similar to humans.

Species selection should be aimed at developing an understanding of the dose-related pharmacology and toxicity profile enabling the selection of a clinical starting dose that is safe and has the potential of providing a benefit to the patient. The pharmacologic active dose range that is predicted by nonclinical data frequently over or under predicts the human response. Consequently, consideration should be given to designing the nonclinical program to define an adequate safety margin that enables dose escalation in humans that is greater than what is predicted by nonclinical pharmacology models.

Understanding dose-related differences in biodistribution profile between species is an important first step in species selection (Table 1, Guidance P, X). Studies with the therapeutic candidate or representative construct (tool transgene/surrogate) can be used to justify the species selection for definitive studies that establish the pharmacologically relevant dose range and toxicity profile. If these preliminary studies reveal distinct differences between species in the biodistribution profiles, then consideration should be given to which species will most likely predict human response. Data used to select species for definitive studies should include a comparison to nonhuman primate (NHP) unless there is relevant human data. If there are distinct differences between NHP and other species and the decision is made not to use NHPs, then consideration should be given as to how this difference will be factored into the clinical trial doses. If available, data from humans using a similar vector and dose can also be used in justifying the relevant animal species and may support the use of species other than NHP.

The use of *in vitro* data comparing species differences in cellular tropism and transgene expression may also aid in justifying the most relevant species. However, caution should be used in relying on these data exclusively as *in vitro* transduction efficiency and relative tropism may not replicate that observed in *in vivo* studies.^9^

Single species toxicology programs are acceptable with the intent to use the species that most likely predicts the human dose-related pharmacology and toxicity response. There may be situations where there is no relevant species, such as an expressed protein that is not pharmacologically active/expressed in normal animals or that generates a species-specific immune response or an expressed nucleic acid that has homology-dependent activity specific to humans. Additionally, therapies targeted to treat viral infections or human-specific mutations that do not exist in animals may not have a pharmacologically responsive model. In such situations, scientifically justified alternative approaches should be considered. Animal-specific surrogates, engineered animal models or disease model systems, and immune suppression to enable administration or engraftment of human-specific products can be used to address animal-specific pharmacology or immune responses.

For viral targets or human-specific mutations, *in vitro* models can be useful to evaluate human-specific responses, but ideally concordance with animal studies to evaluate safe doses with the appropriate level of the gene product delivered to the target tissue should be considered.

### Immunogenicity assessment

Immunogenicity assessment is typically included in gene therapy toxicology studies, with the intent of understanding the potential immune response to the transgene-derived product to better characterize the transgene-derived product exposure profile to enable safety evaluation (Table 1, Guidance N, AK). Most often the clinical gene therapy candidate and human transgene-derived product is evaluated in animals, which can induce a species-specific immune response to the foreign human protein in animals, of which the relevance to human risk assessment is limited. The immunogenicity risk of a transgene-specific product to patients is thus best evaluated in clinical studies.^10^

### Genomic risks: viral insertion and genome editing

Gene therapies pose distinct potential risks to the genome, including insertional mutagenesis (from integrating viruses), mutation (from genomic editing), and altered (off-target) gene expression (from promoters used to drive transgene expression). Table 2 summarizes key nonclinical safety considerations to highlight the general expectations from regulatory agencies for gene therapy products to guide the nonclinical program design; these are neither exhaustive nor prescriptive. Two major classes of gene therapies have emerged that have helped to define our current approaches to integration risk, viral vectors, and direct genomic modification.

**Table 2.** Considerations for the safety assessment of viral vectors

Topic and Guidance Ref.	Considerations for the Safety Assessment of Viral Vectors
Replication competency ^2,50^, Table 1—Guidance L, N, Y	If vector is replication competent, what is the therapeutic rationale and implications to the patient? What are risks to close contacts/health care providers from accidental exposure or shed virus? Does the shed or released virus pose an environmental risk? Does patient treatment or immune status affect risk of local/systemic viremia? If vector is not intended to be replication competent, are replication-competent viral particles present as an impurity during manufacture? Have nonessential accessory genes been removed or other engineering/manufacture steps been taken to minimize risk that recombination could lead to replication competency (*e.g.,* low sequence homology, use of self-inactivating vectors, separation of vector sequences onto multiple plasmids)? For oncolytic viruses, demonstrate tumor selectivity.
Viral strain/serotype^2^	What is the derivation of the parental viral strain/serotype used for the vector? Is it a passaged strain or novel isolate? How does this affect potency/viremia or other viral properties? Are there known liabilities from infection by the (wild-type) viral vector (including risks to pregnancy and immunocompromised patients)?
Engineering/attenuation ^2^, Table 1—Guidance L, Y	What genetic elements comprise the therapy and what sequence modifications have been made to the parental viral strain? How do these affect replication competency, relative potency, biodistribution/tropism, cell-specific replication, or interactions with host immunity? Does vector transgene lead to expression of a nontolerated or immunogenic protein/oligonucleotide? Is expression local/systemic, acute/chronic?
Recombination ^2^	If viral vector is replication competent, what are possible risks from novel strains arising from recombination with wild-type viruses (or itself)? If vector is not intended to be replication competent, what modifications to virus or product manufacture are taken to minimize risk of recombination (*e.g.,* separation of viral components onto distinct plasmids, incorporation of self-inactivating designs)? Are there homologous sequences in the genome that generate ‘hot spots' for recombination?
Complementation	If virus has been attenuated, can other viruses complement for the deficiency to restore virulence?
Tropism (biodistribution) ^2,50^, Table 1—Guidance N, Y	What is the natural tissue tropism of the parental viral strain? Does viral engineering alter tropism (*i.e.,* pseudotyping) or mechanisms of infection? If so, how does this potentially impact biodistribution/safety? Is there evidence of vector in germ cells or of transgenerational gene transfer (or integrated vector in germinal DNA)? How is the virus cleared and what are the routes secretion/excretion that could give rise to secondary exposure?
Host range ^2,50^, Table 1—Guidance L, Y	What species can be infected with the virus? Does this pose an environmental risk from viral shedding/accidental release? Does species host range support use of selected species for pharmacology, biodistribution, or safety assessment? Is this supported by evidence of transfection, transduction, infectivity, and/or replication in cells/tissues of the selected species? Do genetic elements or expressed proteins of the transduced vector function in that species (*i.e.,* promoter or regulatory elements, expressed protein/oligonucleotide)? Are there significant differences in transduction efficiency between species and how does this impact considerations for translation of the therapeutic dose–response and safety margin to humans.
Vector copy number ^2^	How many copies of the transgene are incorporated per target cell? Does the safety program support the copy number range? Note: Standards for acceptable vector copies per cell have not been formalized, but for retroviral vector levels, <5 are generally accepted by regulatory agencies for *ex vivo* engineered cell products.^17^
Genomic integration, genomic risk, insertional mutagenesis ^2^, Table 1—Guidance Y, Z	Does the virus recombine or integrate with the host genome? [Note that episomal vectors, such as AAV, have low-frequency integration events that are prompting regulatory expectations for these assessments.] If so, is the observed integration profile similar to that reported for the wild-type viral strain? Do the sites of integration disrupt cell/gene regulatory mechanisms? Could these integration events promote mutagenic transformation (*e.g.,* insertional disruption, insertion of promoter sequences) or cause other forms of genomic risk (*e.g.,* breaks, recombination)? Provide detailed analytical and bioinformatic methods for regulatory review.
Availability of antiviral therapy	For replicating virus, are antiviral therapies available in case of a virus-related adverse effect or accidental exposure? Has viral engineering altered viral response to antiviral therapy?
Viral load, persistence, and shedding ^2,50^, Table 1—Guidance L, N	For replicating viruses, what is the viral burden over time? Is viral replication dependent on patient-specific factors (*e.g.,* tumor load, immune status, comedication, age)? What is the persistence of the virus in the patient (or nonclinical model)? For replication-competent viruses, what are the routes for viral shedding? What precautions are required to minimize environmental release or exposure to close contacts? How long are precautions required to protect from exposure to shed virus?
Latency/reactivation ^2,50^, Table 1—Guidance L, N	Is there evidence that the virus can undergo a latent infection, with subsequent reactivation/viremia? Does viral engineering, including attenuation, alter this risk? How does latency/reactivation affect shedding risk? Are nonclinical models available to assess risk of latency/reactivation?
Special populations ^2^	For attenuated or replication-competent viruses, does disease state or host immune status confer differential risk? Should special precautions be warranted for coadministration of agents that suppress or alter immunity? For both replication-competent and defective viruses, are there risks to pregnancy or of maternal/fetal transfer?
Translational systems ^2^, Table 1—Guidance L, Q	Are patient-derived or engineered animal cells, or disease animal models (natural or genetically altered) available that can inform pharmacology proof of concept, safety, and/or dose selection? Can alternative/surrogate viral serotypes vector constructs facilitate pivotal nonclinical pharmacology or safety studies as compared with the intended human clinical product?

AAV, adeno-associated virus.

### Insertional mutagenesis with viral vectors

From a toxicology perspective, the site of viral integration can pose a genomic risk, resulting in cellular dysregulation and, at worst, transformation. This transformation can be attributed directly to the disruption of genes controlling cellular proliferation, or indirectly by integration of vectors harboring an internal promoter that alter expression of neighboring genes. The potential risk of transformation is further shaped by the promoter, transgene, and other aspects of vector design and the number of vector copies inside a cell. In the case of replication competent vectors with the potential of genomic integration (*e.g.,* oncolytic viruses), insertional mutagenesis risks would increase as the viral load increases. Except for HSV-1, which is a nonintegrating virus and so not further considered here, specific considerations for insertional mutagenesis risk assessment for approved genetic vector are discussed below and in Table 3.

**Table 3.** Viral vector genomic integration and insertional mutagenesis risk

Virus Type	Genomic Integration and Transformation Risk	References
Gammaretrovirus	Insertional oncogenesis observed with *ex vivo* retrovirally mediated gene transfer, using Moloney murine leukemia virus, of the interleukin-2 receptor γ-chain gene into CD34^+^ cells in patients with severe combined immunodeficiency. Insertional mutagenesis involving *LMO2* and identified the potential involvement of other proto-oncogenes, including *CCND2* and *BMI1*. Genomic integration bias in and near gene coding regions and around transcription start sites.	^13^, Table 1—Guidance P, X
Lentivirus	HIV-associated cancer risk is attributed to reduced cancer immune surveillance and not to virally mediated insertional oncogenesis. Integration biased to active transcription regions and genomic regions enriched in G/C bases, near transcription-associated histone modifications, and genomic regions structurally associated with outward-facing DNA major grooves. A consensus nucleotide-enrichment pattern around the integration sites has been established.	^20,23–25^
AAV	Generally considered an episomal virus, although viral integration occurs at a low frequency (∼0.1–0.5% integrations per AAV infectious unit) and is generally random. Because rAAV vectors are deficient in the Rep gene, they lack the targeted integration capacity of wt AAV, which typically integrates in chromosome 19, at actively transcribed genomic regions and regions associated with DNA breaks. DNA breaks from CRISPR/Cas gene editing also provides sites for AAV integration.	^31^, Table 1—Guidance B, AC

Cas, CRISPR-associated; CRISPR, clustered regularly interspaced short palindromic repeats; rAAV, recombinant AAV; wt, wild type.

Clinical observations of insertional oncogenesis have been documented following the use of a Moloney murine leukemia viral vector, a gammaretrovirus, in the *ex vivo* transduction of CD34^+^ stem cells to express the IL-2 receptor γ-chain gene. This outcome spurred vector design and transduction strategies to minimize oncogenic transformation. One such strategy utilized a modified self-inactivating gammaretrovirus vector for severe combined immunodeficiency, which did not result in a measurable change for insertional mutagenic risk.^11^ Such self-inactivating viral vectors, which harbor mutations in the enhancer and promoter regions within the viral long terminal repeats, reduce insertional oncogenesis and cell immortalization risks^12^ without altering genomic insertional preferences.^13^ In addition, promoter/enhancer selection in vector design has been proposed to influence transformation risk,^14^ as recently suggested in a lentiviral clinical trial for cerebral adrenoleukodystrophy.^15^

AAV-associated insertional mutagenesis linked with hepatocellular carcinoma (HCC) was reported in neonatal mucopolysaccharidosis type VII mouse following intravenous injection of an AAV2-based gene therapy vector.^16^ Initial studies suggested that the increase in HCC was limited to neonatal mice^17^ or juvenile mice, but subsequent investigation demonstrates that AAV integration is also associated with development of HCC in adult mice and not an age-dependent risk.^18^ Insertional site analyses mapped a common AAV integration site in the *Mirg* and *Rian* gene loci on chromosome 12 in mouse, which is associated with numerous regulatory RNA sequences.^19^ Accumulation of AAV integration into the *Rian* locus and increased HCC incidence in mouse models were subsequently replicated, with increased risk linked to hepatic inflammation, hepatocellular turnover, vector design, and dosing.

Clonal expansion has also been observed in dogs treated with an AAV8 or AAV9 vector expressing canine factor VIII attributable to genomic integration,^20^ but a similar 10-year AAV-FVIII dog study noted hepatic AAV vector integration rates lower than the spontaneous human mutation rate.^21^

Despite these findings in mice and dogs, there have been no confirmed associations between exposure to an AAV-based gene therapy vector and insertional tumorigenesis in humans or nonrodent models. Moreover, the prevalence of HCC in humans is 10 per 100,000^22^ despite a relatively high AAV-seroprevalence rate (∼30–50% for AAV2^23^), and linked other factors, including hepatitis C virus, indicating a low penetrance for AAV-associated HCC if there is a causal relationship.

A decade-long review of gammaretroviral chimeric antigen receptor (CAR) T cell safety and function revealed no evidence of vector-induced immortalization of cells, no evidence of clonal expansion, and no enrichment for integration sites near genes implicated in growth control or transformation.^24^ In contrast, clonal T cell expansion is documented for naturally occurring HIV integration targeting the transcriptional regulators MKL2 or BACH2,^25^ and for lentivirally transduced engineered T cells associated with integration proximal to *TET2*^26^ and *CBL*,^27^ but these were deemed to arise from selective growth advantage of these T cells clones and not transformation.

### Viral integration site assessment and cancer risk

Viral integration can pose genomic risk associated with gene disruption, altered gene expression (directly or through proximity of promoter elements), or creation of genomic breaks/fusions. Careful assessment of insertional mutagenesis is warranted to establish the strategy for assessing genomic injury. Sequencing-based methods have evolved to assess both genomic and transcriptomic alterations arising from viral (and other) transduction systems, including validated commercial methods for mapping insertional preferences. Typically, these methods rely on polymerase chain reaction (PCR) detection of unique viral or transgene sequences, characterizing neighboring genomic sequences to enumerate the depth and range of genome-wide insertion events.

Application of bioinformatic methods allow for characterization of nucleotide-specific biases proximal to the integration site, as well as characteristic features such as chromatin state, methylation status, proximity to transcriptional start/end sites, CpG-rich regions, and insertional frequency toward specific genes, including proximity to oncogenes/tumor suppressor genes. Unless a functional consequence is demonstrated, there has been no association of oncogenic risk associated with a specific integration profile.

Similar to biologics, an evaluation of potential carcinogenesis should be performed on a case-by-case basis. Interspecies differences in viral tropism, vector design, promoter usage, and other factors generally limit the utility of standard rodent models for carcinogenicity testing, and regulatory agencies do not currently require these before clinical evaluation. The induction of HCC in mice with high-dose systemically administered AAV vectors is well documented over the last 20 years and should not need to be repeated for most other AAV candidates. However, if the transgene to be expressed has potential for growth acceleration, for example, evaluation of hyperplasia and clonality may be warranted; viral biodistribution and transduction information can be used to target pathology assessments. Should hyperplasia be observed, specific integration site mapping can be used to inform an assessment of insertional mutagenesis. *In vitro* systems could assist in understanding the relative risk of unwanted proliferation.

Such methods can complement more traditional approaches for assessing transformation risk, including longer-term *in vivo* studies to assess tumorigenesis or *in vitro* methods to assess transformation potential. However, no consensus in the scientific or regulatory community exists regarding how best to characterize insertional mutagenesis/transformation risk, and substantial challenges remain in designing/validating either *in vitro* methods (*e.g.,* IL-2 independent growth of CAR T cells^28^) or *in vivo* methods.

Given the breadth of issues and unique predilections of specific viruses, transgenes, promoters, and target cells/tissues, a strong weight-of-evidence analysis regarding the context of use must be conducted to inform and characterize risk. These can form the basis for regulatory interactions to define credible measures for informing patient risk and safety.

### Genome editing

Genome editing also poses a genomic risk. These technologies are being used to correct mutated genes, inactivate genes, delete genes, or insert genes into specific genomic sites in somatic cells. Several clinical studies are evaluating genome editing technologies, including zinc finger nucleases, meganucleases, transcription activator-like effector nucleases (TALENs), a combination of meganucleases and TALEN, and clustered regularly interspaced short palindromic repeats (CRISPR)/CRISPR-associated platforms.

These engineered nucleases are designed to produce site-specific double-strand breaks (DSBs) in the genome,^29^ and the consequences depend on two main DSB repair pathways, including nonhomologous end joining (NHEJ) and homology-directed repair (HDR). NHEJ, the most common pathway, is imperfect (error-prone) resulting in small insertions and deletions (indels) of nucleotides at the cut site, which can lead to disruption of the target gene (or regulatory sites) by creating frameshift mutations that can result in expression of a truncated and/or nonfunctional protein.^30^ HDR is a repair process that uses a homologous donor DNA template for repair of the DSB and is essentially error free.

The donor DNA template can be used to either introduce or correct a mutation in a specific gene locus or insert a corrective DNA fragment or entire gene into the DSB cut site. Some of the specific risks associated with genome editing include off-target editing, potential effects of on- and off-target editing as well as the unknown long-term consequences of editing.

Genome editing introduces risks for potential off-target genome modifications that can lead to aberrant gene expression, chromosomal translocation, potential malignancy, insertional mutagenesis when integrating or nonintegrating vectors are used to deliver the genome editing components, and the associated risk of tumorigenicity; and/or the possibility of an immune response to the genome editing components or the expressed transgene (Table 1, Guidance P).

Genome editing products share common features with gene therapies, including nucleic acid delivery, biodistribution assessments, transgene and protein expression, and persistence, thus regulatory guidance on these nonclinical aspects of gene therapy development can be incorporated into safety assessment of genome editing products (Table 1, Guidance A–C, P, X). FDA guidance (Table 1, Guidance X, AC) provides a road map for development of genome editing products based on the technology used to edit the genome, target cell type to be edited, the degree of genomic modification and delivery of genome editing components to target cells. Regarding editing technology, the specificity, selectivity, and efficiency of editing at the target site need to be demonstrated, as well as determination of the level of genome modification needed for desired therapeutic effect.

Based on the generation of DSB, genotoxicity risk is a key focus for safety evaluation of genome editing therapies, which includes evaluation of unintended nuclease activity at off-target sites within the genome and potential impact of this activity as a long-term or permanent genetic modification of patient cells.

Several orthogonal methods are used to assess genotoxicity risk, including bioinformatics, biochemical, molecular, and cell-based assays. A gold standard method for identifying all off-target sites has not been determined, however, several unbiased, genome-wide and sensitive methods are commonly used.^31^ These methods are referred to as unbiased since DSB are detected without any assumption of type of off-target site and include integration-defective lentiviral vector, oligonucleotide capture, GUIDE-Seq, CIRCLE-Seq, and CHANGE-Seq.^32,33^ Once an off-target site is identified, a risk assessment is conducted for the edited off-target gene/loci, location of the cut site, likelihood of expression of the edited gene in target and nontarget tissue(s) and potential downstream effects of editing (*e.g.,* truncated protein, impact on tumor suppressor or oncogene).

Selection of orthogonal methods to assess genotoxicity is based on the type of engineered nuclease and nature of the edited product. For genotoxicity assessment supporting *ex vivo* editing, karyotyping is conducted to assess potential structural and/or numerical chromosome aberrations, including translocations. A molecular translocation assay designed to identify all possible translocations associated with on- and off-target editing activity and potential impact to human health (through bioinformatics and literature searches) is part of the genotoxicity risk assessment. For *in vivo* editing programs, the tumorigenicity risk can be tested *in vitro* in cell-based assays such as the soft agar transformation assay,^34^ however, regulatory guidance is not yet available to specifically recommend a comprehensive set of assays for genotoxicity testing for genome editing technologies, likely due to the rapid advancement of new technologies for assessing potential genotoxicity.

Persistence of genome editing components should be considered in risk assessments, for example, delivery of genome editing components as mRNA to cells *ex vivo* or through lipid nanoparticles (LNPs) *in vivo* will have a short duration of editing activity compared with editing components delivered through viral vectors with much longer persistence.

For *in vivo* editing programs, the number and choice of species need to be justified as relevant for safety evaluation. Hybrid pharmacology/toxicology studies can be used to establish proof of concept in an animal disease model as well as evaluate safety.^35^ Target tissues can be evaluated for editing efficiency (% indels), vector genomes, transgene expression, and anticipated pharmacodynamic response, which allows understanding of degree of editing needed to provide adequate response in animals and possibly patients.

The traditional battery of genotoxicity assays used for small molecule development is not appropriate for evaluating genome editing technologies as nucleic acids encoding the engineered nucleases are delivered *in vivo* using AAV vectors or through LNPs. Cellular expression of the engineered nuclease is thus needed for assessment of genotoxicity risk, whereas traditional genotoxicity assays rely on direct administration of a compound to a well-validated battery of *in vitro* and *in vivo* assays. Traditional genotoxicity methods can still be used to assess certain impurities or components of the delivery system (*e.g.,* lipid components of LNPs). Similarly, species differences in viral tropism, DNA/RNA sequence homology, and study power/statistical sensitivity constrain the practical utility of long-term (12+ month) or 2-year carcinogenicity bioassay studies. These limitations constitute real barriers to the conduct of chronic toxicity and carcinogenicity studies, which will require case-specific discussions between industry and regulatory agencies.

The diversity of integrating and nonintegrating vectors, in addition to direct genome editing modalities, underscores the importance of case-by-case flexibility to assessing their potential safety liabilities. However, extensive nonclinical and clinical experience can serve as the basis for alignment on general considerations and approaches for identifying potential hazards, focusing on relevant study designs and endpoints to inform clinical risk/benefit.

## Biodistribution/shedding

Gene therapy biodistribution studies assess the DNA of the virus and/or gene therapy location, where the transgene product is expressed, persistence, and potentially the routes of shedding applied to tissues and biofluids over time.

Additional shedding studies evaluate risks of secondary exposure (*e.g.,* health care workers, contacts) and environmental exposures for products that use replication-competent viruses (Table 1, Guidance K, Y). Whereas standard biodistribution studies may use sensitive molecular methods for detection of viral DNA/RNA, such methods may not inform infectious potential from intact viruses. For example, shedding studies may consider methods to determine infectious potential where vector is replication competent or carrying a dangerous transgene. Otherwise, simple detection for the presence of vector sequences should suffice. In addition, for certain viruses, such as HSV-1, which have an established risk to offspring from transfer of maternal wild-type infection in late gestation or during birthing, additional assessments of biodistribution in the context of pregnancy models may be warranted, as described in the Imlygic (talimogene laherparepvec) FDA approval documents.

Gene therapy biodistribution data are analogous to pharmacokinetic data generated for pharmaceuticals or biotherapeutics commonly incorporated into pharmacology and toxicity studies. Biodistribution data can be important in selecting the most appropriate species, designing pivotal nonclinical pharmacology and safety studies, and developing a clinical safety monitoring strategy. Preliminary biodistribution studies can characterize the dose-related distribution of the delivery vehicle/vector and may include a reporter gene instead of a therapeutic transgene, which are important for guiding species selection. Published and historical data can minimize the need for preliminary or extensive biodistribution studies. Some gene therapies use a cell-specific promoter, and expression data from biodistribution studies can then be important in confirming the specificity of the promoter activity.

Pivotal biodistribution studies typically include a list of tissues and biofluids as indicated in the draft ICH S12 and this list may be augmented based on experimental or historical data, and may include tissues of special interest (*e.g.,* dorsal root ganglia for AAV) or for assessing pharmacology. The tissue list may be reduced if there are existing data from the innovator on the same vector (viral vector serotype, or nonviral vector of the same composition), made by a manufacturing process producing material of a similar quality and administered at a similar dose and route of administration. If a device will be used in patients to deliver the vector, then nonclinical studies will need to incorporate a similar device, if feasible.

The methods used to evaluate vector in tissue and biofluids will primarily evaluate the vector DNA and potentially the expressed transgene through mRNA and/or protein. Standard quantitation methods based on PCR are typically used for assessing vector related nucleic acid. Regulatory guidance indicates that PCR assays should be qualified to detect 50 copies of vector DNA per microgram of genomic DNA. Evaluation of protein expression from a vector may include activity assays, antibody-based detection methods or mass spectrometry-based assays. Frequently, these assays will need to be designed to differentiate between the endogenous gene or its product and the vector-derived material. While the biodistribution assays need to be appropriately qualified and documented, there is no regulatory expectation that the assays are conducted under GLP guidelines.

Biodistribution studies will typically include a minimum of two time points with the early time point chosen to approximate the expected time of initial maximum expression of the transgene and one additional time point that enables an assessment of persistence of the vector's DNA and transgene expression. In addition, for certain viruses, the selection of later time points may inform risk of reactivation of latent viral infections, which may necessitate analytical methods to detect infectious particles (as some PCR-based methods may only assess viral fragments).

Animal numbers for biodistribution should be sufficient to draw a valid scientific conclusion on the tissues that a vector distributes to, and relative abundance of the vector DNA and transgene expression between tissues. If the biodistribution data are being used to interpret toxicology findings or design safety monitoring strategies in patients, then the highest dose tested in the toxicology study will typically be evaluated. Lower dose levels may also be evaluated to understand how tissue concentration of the vector DNA and transgene relate to toxicity and the therapeutic index.

Depending on the vector and disease indication, the assessment of vector DNA integration into host cell DNA may be needed to evaluate the potential germline transmission or to evaluate genotoxicity and carcinogenic risk. If these endpoints are not a specific endpoint in a study, consideration should be given to collecting and retaining tissue samples to enable integration assessment at later stages of product development if requested by regulatory authorities.

These concepts in this section are currently under discussion as part of the first harmonized ICH guidance for gene therapies, ICH S12.

## DEVELOPMENTAL AND REPRODUCTIVE TOXICOLOGY SAFETY TESTING

The product type, mechanism of action, biodistribution, shedding profile, and target patient population should be considered when assessing the potential for developmental and reproductive toxicity (Table 1, Guidance C, X). A primary concern involves the potential for inadvertent germline transmission, which is dependent on the nature of the product; however, this can be influenced by the route of administration and dose level. Products that comprise nonreplicating plasmid DNA, nonreplicating viral vectors, nonintegrating viral vectors, or lack tropism for germ cells have a lower risk of germline transmission limiting the need for developmental and reproductive toxicology (DART) studies. However, replicating viruses with integrating capacity and tropism for germ cells are of higher risk. The risk of inadvertent germline transmission can be addressed by vector biodistribution studies showing both lack of expression and activity in the testes or ovaries. Semen clearance or sperm fractionation studies can be conducted, if necessary, to demonstrate lack of transduction of spermatozoa.^36^

If there is no detection in gonadal tissue or no detection in germline cells (*e.g.,* sperm, oocytes), then further DART studies and patient monitoring to assess germline transmission are generally not warranted. This approach is reinforced by experimental evidence showing that there is no evidence of AAV-based germline transmission to date.^37,38^ If there is detection in germline cells, rodent breeding studies may be required.

The mechanism of action of the product should also be evaluated for any target-specific concerns that may warrant further evaluation of DART. In many cases, the target-related evaluation can be made based on available scientific literature without the need for DART studies; however, there may be cases where the existing information is insufficient to assess DART and embryofetal development studies to assess placental transfer and the effects of transgene expression may be useful to fully inform risk if the targeted patient population includes women of child-bearing potential.

### Juvenile/pediatric safety considerations

As with DART, the need for juvenile toxicity studies should be based on a weight of the evidence approach to determine if directed studies will add to the risk assessment. In general, if clinical trials can be started in adult populations and then bridged to younger patients, juvenile toxicity studies should not be required unless there is a specific concern that cannot be addressed with the existing data. In this case, a directed juvenile toxicity study targeting the areas of concern may add value to the risk assessment. However, if the initial clinical population involves infants or young children, the use of juvenile animals in the initial toxicology studies should be considered, especially if the distribution of the vector is thought to vary with relationship to age.

## DOSE TRANSLATION: NONCLINICAL BASIS FOR HUMAN DOSE SELECTION

Human dose selection for first-in-human (FIH)/first-in-patient (FIP) gene therapy clinical studies takes into consideration the same nonclinical principles used for other medicines with the caveat that translation between species and to humans for gene therapy medicines is not well understood. This uncertainty stems from species differences associated with target cell transduction and transgene expression efficiencies. Regulatory guidance allows for creativity and nonclinical programs designed on a case-by-case basis since one prescribed manner of selecting the FIH/FIP dose would not be applicable to the many different types of gene therapies under development (Table 1, Guidance B, Z).

Given ethical considerations, FIH studies conducted with gene therapies will be done in patients and not healthy volunteers. Viral vectors are typically only administered a single time, due to development of an immune response that can neutralize the effectiveness of subsequent vector administration. Consequently, dose escalation in an individual is not possible and the starting clinical dose should be selected to provide a benefit to the patient. Nonviral vectors, which typically do not induce a neutralizing immune response, may be administered multiple times and consequently allow dose escalation within the same patient.

Nonclinical studies also need to define the anticipated maximum efficacious dose. Dose selection should consider the potential for toxicity associated with the delivery vector and/or the transgene product and ensure that the gene therapy can be administered safely within the anticipated therapeutic dose range. Because of the uncertainty in how the nonclinical dose repose will translate to patients, the nonclinical safety studies should provide a safety margin that enables doses greater than what the nonclinical pharmacology models predict will be the efficacious dose range.

Additionally, there can be threshold effects where a relatively small change in the dose results in a disproportional increase in transgene expression.^39^ Ideally, a safety margin that is appropriate for the disease indication and potential adverse effects should incorporate the potential for nonlinear dose response. For indications that have high unmet medical need, are severely debilitating, and/or life threatening, minimal or no safety margin may be acceptable. Like other therapeutic modalities, the nonclinical studies will establish a safety margin and characterize potential adverse effects so that appropriate monitoring strategies can be developed for the clinical trial.

Nonreplicating vector gene therapies are typically administered based on vector genomes per kilogram, organ, or compartment volume or as an absolute dose. For intravenous-administered gene therapies, vector genomes per kilogram is typically used. When administering to a confined anatomic compartment, then volume of that compartment is typically used (*i.e.,* cerebral spinal fluid). When delivered through intraparenchymal infusion to specific target sites in the brain, dose level can be based on exposure of specific region (*e.g.,* including dose, dose volume and volume of transgene expression at target site) or hemisphere. A fixed absolute dose is appropriate for the subretinal space of the eye. Note that for replicating viral vectors, the dose may be administered based on plaque-forming units per kilogram. Once the relevant dosing parameter is determined, then an understanding of the dose required to correct a disease phenotype in an appropriate disease model needs to be determined. This dose may scale directly to the human dose.

However, if the interspecies comparisons of dose-related biodistribution indicates that the transduction efficiency in the disease model may be distinctly different than human, then an approach to incorporating interspecies differences in the human dose prediction should be considered. If the disease model is a rodent and another species more closely mimics the human transduction efficiency of the vector, then consider a study to identify a dose that gives similar transgene expression in the rodent for correcting the disease phenotype.

Once the therapeutic dose range is established, doses for toxicity studies are typically selected to bracket the efficacious dose range with the high dose either being the maximum feasible dose or a dose that provides an appropriate multiple to the anticipated maximum efficacious dose. Other dose selection strategies may be appropriate, but typically the low dose is selected to match the anticipated clinical starting dose and the mid dose the anticipated maximum dose that will be used in the clinical trial. Toxicity studies that evaluate two dose levels may also be acceptable. These will typically be done when there is high confidence that the starting dose is safe in humans. The doses selected for these studies will typically be the anticipated maximum efficacious dose and a greater dose that will evaluate an acceptable safety margin.

With an understanding of the therapeutic dose range, the types of adverse effects that may be observed, the safety margin and an appreciation of the uncertainties in translating nonclinical data, the clinical starting dose, dose escalation, and safety monitoring strategy can be developed. This will be done on a case-by-case basis in consultation with regulatory authorities.

### Study design considerations to enable clinical redosing

One general limitation of many gene delivery and editing approaches is the inability to redose or titrate the therapy in patients. The inability to redose is driven by host immunity mounted against the initial therapeutic intervention, but similar limitations can even preclude patients from the therapy entirely based upon preexisting antibodies following environmental exposures.^40^ Several approaches are being explored to circumvent the challenge of immunogenicity, which include plasmapheresis, decoy therapies, coadministration of immune modulators to suppress induction of anti-capsid humoral, IgG-degrading enzymes (*e.g.,* imlifidase), and cell-mediated responses.

Other approaches leverage platforms that are unrecognized by the immune system, such as nonviral-based gene delivery through LNPs. If successful, gene therapies can evolve to be more inclusive of patients currently excluded by preexisting antibodies, provide patient-specific dose titration, address concerns about therapeutic durability through redosing, and enabling pediatric/juvenile therapeutic intervention even for targets in mitotically active tissues (*e.g.,* liver).

Nonclinical studies would need to incorporate aspects of repeat dosing to enable the clinical dosing paradigm, accounting for redosing to achieve initial therapeutic titration and periodic boosting of the transgene within the context of existing toxicology study designs. These initial titration steps would likely provide biphasic risks, an acute phase where the delivery vehicle was present and a second phase where accumulation risks from a nucleic acid payload would be of primary concern.

A repeat-dose toxicology study design could model this with dose levels and dosing frequency greater than or equal to the clinical program, likely in parallel to single-dose groups. In the therapeutic booster design, lifetime dosing in toxicology studies is likely unnecessary and unwarranted since the objective is to maintain a therapeutic window of exposure. Monthly repeat dosing as part of the existing toxicology study may be adequate to model this booster effect, provided it overestimates the clinical risks of accumulation. A solid understanding of the pharmacokinetic and biodistribution profiles during dose titration and boosting paradigms would likely inform the appropriate toxicology design to ensure exposure coverage in the clinical setting.

## FUTURE CHALLENGES FOR THE NEXT GENERATION OF GENE THERAPY PRODUCTS

Next-generation gene therapy modalities are in early stages of nonclinical development to address current limitations of gene therapy products. Researchers are devising novel ways of engineering AAV capsid proteins to target other tissues besides liver more effectively or to cross the blood–brain barrier and target regions within the central nervous system following intravenous administration. Advances in genome editing continue to selectively disrupt a target gene or allow incorporation of cDNA encoding a gene of interest into the nuclease-induced cut site. New molecular tools are continually evolving to better assess levels of on- and off-target nuclease activity. Next-generation editing technologies that result in selective excision of nucleotide bases without inducing DSB include base editing,^41,42^ prime editing,^43,44^ and peptide nucleic acid-based editing.^45^ Additionally, site-specific recombinases promote breaking and rejoining of DNA strands at specific targeted sequence positions.^46^

Nonclinical safety assessment strategies will also evolve in parallel with next-generation gene therapy modalities. Challenges include determining human health risk to genome editing and base editing as methods become more sensitive to detect off-target sites, developing novel tools to assess off-target base and genome editing and best practices to assess genotoxicity risk. For gene regulation therapies, the ability to tune to the appropriate level of repression or activation will be paramount to the success of these therapies, and methods to assess impact of on- and off-target gene repression or activation will continue to evolve. Much of the same nonclinical assessment strategies for biodistribution analysis, toxicology evaluation, reproductive/developmental safety, and tumorigenicity risk assessment will be used for these novel technologies. However, the uncertain aspects of these next-generation gene therapies also underscore the need for flexible regulatory guidance, to accommodate the evolving nature of the field.

## DISCUSSION

The challenge of assessing the nonclinical safety for gene therapy products lies in our incomplete knowledge of this evolving field. Study designs and nonclinical models are emerging that have learned from past failures, leading to successful medicines with many more gene therapies on the horizon. This experience has given rise to regional regulatory guidance largely consisting of general considerations to nonclinical safety, acknowledging the unique considerations of each investigational product, and the need to characterize a product's benefit/risk profile for the intended patient populations.^47^

Given the broad therapeutic approach that encompasses gene therapy products and our relatively small dataset of nonclinical or clinical experience, a comprehensive and detailed regulatory guidance approach is unlikely to be appropriate to address all scenarios. This challenge to balance rapid innovation with clear regulatory expectations is not unprecedented, occurring just over 20 years ago during the ICH S6 process. The strengths and weaknesses of a harmonized approach are expertly reviewed by Serabian and Pilaro^48^ for biotechnology-derived pharmaceuticals, with relevant lessons for the field of gene therapy.

To begin, the ICH S12, Nonclinical Biodistribution Considerations for Gene Therapy Products, is an example of where harmonization was an identified need. Accumulated data-driven examples of nonclinical biodistribution for gene therapy products that leverage principles of biodistribution from other modalities contributed to the maturity of this topic.^49^ In addition, methodologies for detecting gene therapies, PCR-based DNA detection techniques are generally universal. Collectively, this topic made the best sense as the first harmonization effort for gene therapy nonclinical guidance. Still, there will likely remain questions related to appropriate species, study powering, and length that remain to be defined on a case-by-case basis, which may lead to aspects of nonspecific guidance. While acknowledging the evolving nature of field, especially as previously unidentified safety liabilities appear in nonclinical and clinical studies, we believe there are a few key areas where recommendations can be made as a starting point for discussion on establishing broader nonclinical guidance.

First, in alignment with expectations on the maximum general toxicology study length necessary for biologics in ICH S6(R1), it appears that studies 6 months in duration are sufficient to identify hazards. For gene therapy modalities that express a therapeutic protein, depending on the pharmacology of the transgene, activity of the promoter, etc., innovators may be able to make a scientific argument that a study 3 months in length would support safe dosing in humans. Of course, if novel issues arise in early screening studies where it is unclear whether such liabilities will resolve over time, innovators may want to take studies out for longer to understand recovery. In the case of genomic editing or gene modulation, 6-month studies may continue to be appropriate in most instances. It should also be considered whether the need to understand recovery is required before FIH studies.

Next, there is clearly more to be learned about the clinical impact of genomic integration of AAV vectors into host cells. However, we believe that the nonclinical exploration into this phenomenon has reached a limit considering the models available and is prime candidate for harmonization. Specifically, the available nonclinical data suggests that long-term toxicology studies (>6 months), 2-year carcinogenicity bioassay studies in rodents, or even multi-year large animal studies provide limited value for modeling human cancer risk given their statistical inability to detect such rare events, combined with known differences in rodent tumor development pathways, and potential immunogenicity effects of long-term expression of human transgene in animals.

In addition, there are ample data in published literature characterizing AAV integration in nonrodent species as well as decades of human experience indicting that the risk of AAV integration-related oncogenic risk is very low. Similar to the conclusion drawn by the EMA on their approval of Glybera^50^ following multiple animal studies, additional genome integration profiling in animal species is unlikely to generate a better understanding of potential risk.

This extensive experience with AAV, to date, can likely act as a blueprint for addressing integration risks as novel AAV based gene therapies emerge. Close clinical monitoring of patients, especially those given AAV systemically, should suffice in mitigating any liabilities in this area. Current guidance from FDA notes that AAV vectors do not possess a significant ability to integrate and thus require “Product-specific” clinical long-term follow-up of “5 years,” while vectors known to integrate with high frequency, such as gamma retroviruses, require 15 years of long-term follow-up. While these concerns of carcinogenicity with AAVs are still being understood and may not automatically default to a 15-year follow-up, we believe that this is a topic that should be aligned across geographic regions, including whether methods such as liver ultrasounds should be required for all recipients of systemically administered gene therapies.

Understanding risks to reproduction and development is a core requirement for nonclinical safety assessments. For gene therapies, addressing this requires a different perspective—given current practice and types of diseases treated by gene therapy, the potential for unintentionally dosing a pregnant woman is extremely low. Rather, innovators need to understand if there is any risk in transducing cells in reproductive organs that may affect fertility or, more worrisome, the genome of sperm or ova. This liability is effectively addressed by a comprehensive assessment of vector biodistribution. Trafficking to the gonads or supportive matrices such as semen has been described in the past, which serves as a key opportunity for harmonization.

Lastly, other areas such as animal numbers and study endpoints (*i.e.,* safety pharmacology) may not require specific guidance at this time. However, harmonized perspectives on the inherent value of large animal models and what organ systems are typically affected from gene therapy platforms would be beneficial to innovators. This in contrast to the expectation of including a usual “core battery” of study endpoints to assess cardiovascular, central nervous system, and respiratory-type organ systems that are usually evaluated for low-molecular-weight drugs and standard biologics.

In summary, an effort to harmonize the overall nonclinical aspects of gene therapies is unlikely to ever be appropriate or inclusive for all topics. Innovators appreciate the case-by-case nature for challenging issues, being able to work with regulators to do what is right for patient safety. However, several key nonclinical safety topics are maturing over 20 years of nonclinical and clinical experience, which could benefit from a harmonized approach, including species selection, certain aspects of study design (*i.e.,* duration), unintended genomic integration risks, biodistribution, and reproductive/developmental studies.

As the ICH process for harmonization is a multiyear process of nomination, drafting, and commenting periods from all concerned parties, we propose that an ICH process to harmonize on the nonclinical safety of gene therapies is now warranted. Given the large number of gene therapy programs in development and in late-stage clinical trials, which will inevitably read out over a multi-year ICH process, the existing and several more years of experience will yield disproportionately greater numbers of examples to ground our approaches or show where our models and assumptions break down.
